# Gut microbiota and child behavior in early puberty: does child sex play a role?

**DOI:** 10.1080/19490976.2023.2278222

**Published:** 2023-11-09

**Authors:** Yangwenshan Ou, Eline Rots, Clara Belzer, Hauke Smidt, Carolina de Weerth

**Affiliations:** a Laboratory of Microbiology, Wageningen University & Research, Wageningen, The Netherlands; b Donders Institute for Brain, Cognition and Behaviour, Department of Cognitive Neuroscience, Radboud University Medical Center, Nijmegen, The Netherlands

**Keywords:** Gut microbiota, microbiota-derived fecal metabolites, child behavior, child sex, puberty, Parasutterella, prevotella 9, absolute abundance

## Abstract

A growing number of studies have indicated relations between the gut microbiota and mental health. However, to date, there is a scarcity of microbiota studies in community samples in early puberty. The current preregistered study (https://osf.io/wu2vt) investigated gut microbiota composition in relation to sex in low-risk children and explored behavioral associations with gut microbiota composition and metabolites in the same samples, together with the potential role of sex. Fecal microbiota composition was analyzed in 12-year-old children (*N* = 137) by 16S rRNA gene sequencing and quantitative PCR. Modest sex differences were observed in beta diversity. Generalized linear models showed consistent behavioral relations to both relative and absolute abundances of individual taxa, including positive associations between Parasutterella and mother-reported internalizing behavior, and negative associations between Odoribacter and mother-reported externalizing behavior. Additionally, Prevotella 9 was positively related to mother-reported externalizing behavior, confirming earlier findings on the same cohort at 5 years of age. Sex-related differences were found in behavioral relations to Ruminiclostridium 5, Alistipes, Streptococcus, Ruminiclostridium 9, Ruminococcaceae UCG-5, and Dialister, for relative abundances, as well as to Family XIII AD3011 group and an unidentified bacterium within the Tenericutes, for absolute abundances. Limited behavioral relations were observed regarding alpha diversity and fecal metabolites. Our findings describe links between the gut microbiota and child behavior, together with differences between child sexes in these relations, in low-risk early pubertal children. Importantly, this study confirmed earlier findings in this cohort of positive relations between Prevotella 9 and externalizing behavior at age 10 years. Results also show the merit of including absolute abundances in microbiota studies.

## Introduction

The human gut is colonized by a great number of microorganisms that are collectively referred to as the gut microbiota. These microorganisms play a critical role in human health.^1^ Gut microbiota composition can be influenced by a wide array of factors, such as age, diet, delivery mode, medication use, health and disease states, geography and socioeconomics, as well as host genetics.^2^ Among these factors, also biological sex is likely to affect the microbiota but has received limited attention in the past.^3–6^ Two large-scale population-level studies (i.e., the Flemish Gut Flora Project and the Human Microbiome Project) observed that sex was moderately related to adult gut microbiota composition.^3,4^ Other adult studies also reported sex-dependent features in gut microbiota composition, such as abundance differences between females and males in *Prevotella*, *Bifidobacterium*, *Akkermansia*, and *Ruminococcus*.^7–9^ Although sex is commonly speculated to impact the gut microbiota from puberty on due to gonadal hormones, its association to microbiota composition has not been well explored in children at this age.^5,6,10^ A recent, albeit small, study found several differentially abundant microbial taxa between pubertal boys and girls, including higher relative abundances of *Alistipes* and *Parabacteroides* in girls.^11^ Therefore, the aim of the current study was to explore sex differences in gut microbiota composition in community children in puberty.

In addition to being related to multiple factors as mentioned above, the gut microbiota, as suggested by a growing body of research, may affect and be affected by brain functions along the microbiota-gut-brain axis (MGBA) in developmentally sensitive time-windows.^12^ Earlier child studies found increased alpha diversity in relation to decreased cognitive ability, fear reactivity, and internalizing problems in infants and pre-schoolers.^13–16^ Regarding specific microbial taxa, the genus *Prevotella* appears to stand out. Loughman et al. observed more *Prevotella* at age one in association with less subsequent internalizing behavior at age two,^17^ while a positive link of *Prevotella* 9 with externalizing behavior was previously reported by us in middle childhood.^18^ It is worth noting that, as sex-specific differences in behavioral problems are often observed in puberty,^19,20^ sex may likely affect microbiota-behavior relations in children at this age.

Despite the fact that the underlying mechanisms of microbiota-behavior relations remain largely unknown, specific microbiota-derived metabolites may be important biological mediators in the complex bidirectional pathways of the MGBA.^12,21–23^ Among these microbial metabolites, short-chain fatty acids (SCFAs), the major colonic fermentation products of indigestible fiber, are thought to influence the communication along the MGBA through immune, endocrine, and vagal pathways.^21,23^ SCFAs, especially butyrate, have been associated with alleviated anxious and depressive symptoms in mental disorders.^21,22^ Branched-chain fatty acids (BCFAs) are commonly formed through the fermentation of protein in the distal large intestine.^23,24^ Despite there only being scarce evidence about the role of BCFAs in the MGBA, higher levels of fecal isobutyrate and isovalerate have been observed in preschoolers with fewer internalizing problems and in adults with depression, respectively.^16,25^ Additionally, another common microbiota-derived fecal metabolite, lactate, might lead to the increases in urine and blood lactate that are seen in depressed subjects; conversely, microbiota-generated lactate may also support hippocampal neurogenesis as a potential anti-depressant molecule.^26^ Based on the above, we also investigated potential associations of the gut microbiota, including its composition and metabolites, with child behavior, taking sex into account.

**Figure 1.**
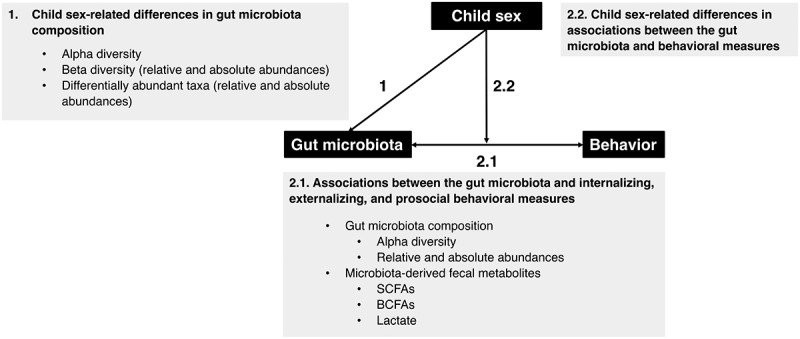
Main research questions of the study. SCFAs, short-chain fatty acids; BCFAs, branched-chain fatty acids.

The present preregistered study (https://osf.io/wu2vt) was carried out on an ongoing longitudinal cohort of low-risk community children when they were 12 years old. The study had two aims: (1) to describe potential child sex-related differences in gut microbiota composition in puberty, and (2) to explore potential associations between gut microbiota composition (i.e., diversity and microbial taxon abundances), microbiota-derived fecal metabolites (i.e., SCFAs, BCFAs, and lactate), and child behavioral measures (i.e., internalizing, externalizing, and prosocial behavior) at this age (Figure 1). Given the scarcity of previous studies in low-risk pubertal children, we did not set up specific hypotheses.

## Results

### Demographics and descriptives

Population demographics and descriptives are presented in Table 1. About 47% (64/137) of the study subjects were girls. Girls showed a significantly higher degree of sexual maturity than boys. Girls also exhibited more child-reported internalizing difficulties and more mother-reported prosocial behavior than boys. No sex differences were observed in other behavioral measures and variables. Finally, child reports reflected significantly more internalizing and externalizing behavior than maternal reports.

**Table 1.** Population demographics and descriptives at the age of 12 years.

		Total *N* = 137	Girls *N* = 64	Boys *N* = 73	Adjusted *p*	
					Wilcoxon test	
Numeric variable		Mean ± SD			Girl *vs* Boy	Child *vs* Maternal reports^†^
Pubertal status		2.74 ± 0.83	2.95 ± 0.84	2.55 ± 0.78	0.02*	-
Age in years		12.71 ± 0.31	12.66 ± 0.3	12.75 ± 0.32	0.16	-
Food Factor 1		0 ± 0.82	0.05 ± 0.83	−0.04 ± 0.81	0.39	-
Food Factor 2		0 ± 0.8	−0.1 ± 0.87	0.09 ± 0.73	0.33	-
zBMI		−0.24 ± 1.09	−0.18 ± 1.01	−0.29 ± 1.17	0.54	-
Child	Internalizing	3.49 ± 2.9	4.41 ± 3.23	2.68 ± 2.31	<.01*	<.01*
	Externalizing	5.27 ± 3	5.75 ± 2.92	4.85 ± 3.04	0.16	<.01*
	Prosocial	8.42 ± 1.38	8.55 ± 1.38	8.3 ± 1.37	0.35	0.83
Mother	Internalizing	2.38 ± 2.55	2.56 ± 2.62	2.22 ± 2.49	0.51	-
	Externalizing	3.42 ± 2.88	2.94 ± 2.58	3.85 ± 3.08	0.16	-
	Prosocial	8.39 ± 1.52	8.91 ± 1.08	7.95 ± 1.71	<.01*	-
**Categorical variable**		**No/Yes**			**Chi-square test**	
					**Girl *vs* Boy**	
Antibiotics		129/8	61/3	68/5	0.86	
Diarrhea		53/84	30/34	23/50	0.29	
Constipation		118/19	53/11	65/8	0.63	

^†^refers to comparisons for the total *N* = 137 children.

*indicates FDR-adjusted *p* < 0.1.

### Child sex-related differences in gut microbiota composition

A total of 186 genus-level microbial taxa were observed using 16S ribosomal RNA (rRNA) gene amplicon sequencing. Furthermore, total microbial abundance was assessed using 16S rRNA gene targeted quantitative PCR (qPCR). We accounted for potential covariates of the gut microbiota (i.e., pubertal status, child age, zBMI, food factors, antibiotics, diarrhea, and constipation) when exploring sex-related differences in gut microbiota composition. No significant sex differences were observed in alpha diversity (as measured by Chao1, Shannon, and phylogenetic diversity indices) and genus-level microbial taxa (i.e., *N* = 84 taxa prevalent in more than 10% of *N* = 137 studied samples), after correcting for multiple tests in generalized linear models (GLMs in Table S1; no multicollinearity problems were observed in GLMs according to Table S2). Beta diversity significantly differed between boys and girls, for both relative abundances (R^2^% = 1.9%, *p* = 0.007, Bray-Curtis dissimilarity) and absolute abundances (R^2^% = 1.10%, *p* = 0.048, Aitchison distance). Variances were homogenous between sexes (*p* = .488 and 0.618 for relative and absolute abundances, respectively).

### Associations between the gut microbiota and behavioral measures

#### Beta diversity and behavior

Only mother-reported internalizing behavior significantly explained variance in gut microbiota composition when using relative abundance data after accounting for potential covariates (R^2^% = 1.79%, *p* = 0.004; Table S3). Absolute abundance data did not reflect such significances.

#### Alpha diversity, relative and absolute abundances, microbiota-derived fecal metabolites, and behavior

We conducted GLMs to assess associations of alpha diversity, relative and absolute abundances, microbiota-derived fecal metabolites with child internalizing, externalizing, and prosocial behavior as reported by either children or their mothers. Considering a lack of consistent criteria in selecting confounders between microbiota-behavior relations, we accounted for different variables in different models (complete GLM results in Table S4, with corresponding multicollinearity tests in Table S5).

Up to 15.48% of microbiota–behavior relations were significant over all the tested models, with more significant relations being identified between taxon relative abundances and mother-reported internalizing behavior, compared to other conditions (Table 2). Remarkably, the numbers of significant relations varied between models and reports, indicating that different additional variables and reporters influenced the significances of microbial predictors. More than one-third of relations were in the same direction using child and maternal reports, and few same-directional relations were significant. Up to 76.19% of microbial taxa displayed the same direction in microbiota–behavior relations between abundance measures. However, few of these same-directional relations were significant for both abundance data.

**Table 2.** Summary of microbiota-behavior relations.

		Model 0^1^		Model 1		Model 2		Model 3	
Significant relations^2^		Child	Mother	Child	Mother	Child	Mother	Child	Mother
Internalizing	Alpha diversity	0/3 (0%)	0/3 (0%)	0/3 (0%)	0/3 (0%)	0/3 (0%)	0/3 (0%)	0/3 (0%)	0/3 (0%)
	Relative	5/84 (5.95%)	9/84 (10.71%)	4/84 (4.76%)	12/84 (14.29%)	6/84 (7.14%)	13/84 (15.48%)	1/84 (1.19%)	6/84 (7.14%)
	Absolute	3/85 (3.53%)	3/85 (3.53%)	3/85 (3.53%)	2/85 (2.35%)	3/85 (3.53%)	5/85 (5.88%)	0/85 (0%)	1/85 (1.18%)
	Metabolite	0/10 (0%)	0/10 (0%)	0/10 (0%)	0/10 (0%)	0/10 (0%)	0/10 (0%)	0/10 (0%)	0/10 (0%)
Externalizing	Alpha diversity	0/3 (0%)	0/3 (0%)	0/3 (0%)	0/3 (0%)	0/3 (0%)	0/3 (0%)	0/3 (0%)	0/3 (0%)
	Relative	1/84 (1.19%)	3/84 (3.57%)	1/84 (1.19%)	3/84 (3.57%)	1/84 (1.19%)	2/84 (2.38%)	1/84 (1.19%)	1/84 (1.19%)
	Absolute	0/85 (0%)	4/85 (4.71%)	0/85 (0%)	6/85 (7.06%)	0/85 (0%)	4/85 (4.71%)	0/85 (0%)	2/85 (2.35%)
	Metabolite	0/10 (0%)	0/10 (0%)	0/10 (0%)	0/10 (0%)	0/10 (0%)	1/10 (10%)	0/10 (0%)	0/10 (0%)
Prosocial	Alpha diversity	0/3 (0%)	0/3 (0%)	0/3 (0%)	0/3 (0%)	0/3 (0%)	0/3 (0%)	0/3 (0%)	0/3 (0%)
	Relative	0/84 (0%)	0/84 (0%)	0/84 (0%)	0/84 (0%)	0/84 (0%)	0/84 (0%)	0/84 (0%)	0/84 (0%)
	Absolute	0/85 (0%)	0/85 (0%)	0/85 (0%)	0/85 (0%)	0/85 (0%)	0/85 (0%)	0/85 (0%)	0/85 (0%)
	Metabolite	0/10 (0%)	0/10 (0%)	0/10 (0%)	0/10 (0%)	0/10 (0%)	0/10 (0%)	0/10 (0%)	0/10 (0%)
**Child vs Mother3**		**Same direction**	**Same direction and sig**	**Same direction**	**Same direction and sig**	**Same direction**	**Same direction and sig**	**Same direction**	**Same direction and sig**
Internalizing	Alpha diversity	3/3 (100%)	0/3 (0%)	3/3 (100%)	0/3 (0%)	3/3 (100%)	0/3 (0%)	3/3 (100%)	0/3 (0%)
	Relative	56/84 (66.67%)	2/84 (2.38%)	60/84 (71.43%)	1/84 (1.19%)	65/84 (77.38%)	4/84 (4.76%)	66/84 (78.57%)	1/84 (1.19%)
	Absolute	64/85 (75.29%)	0/85 (0%)	64/85 (75.29%)	0/85 (0%)	65/85 (76.47%)	0/85 (0%)	66/85 (77.65%)	0/85 (0%)
	Metabolite	7/10 (70%)	0/10 (0%)	6/10 (60%)	0/10 (0%)	6/10 (60%)	0/10 (0%)	7/10 (70%)	0/10 (0%)
Externalizing	Alpha diversity	1/3 (33.33%)	0/3 (0%)	1/3 (33.33%)	0/3 (0%)	1/3 (33.33%)	0/3 (0%)	1/3 (33.33%)	0/3 (0%)
	Relative	62/84 (73.81%)	1/84 (1.19%)	62/84 (73.81%)	1/84 (1.19%)	63/84 (75%)	0/84 (0%)	61/84 (72.62%)	0/84 (0%)
	Absolute	64/85 (75.29%)	0/85 (0%)	63/85 (74.12%)	0/85 (0%)	61/85 (71.76%)	0/85 (0%)	61/85 (71.76%)	0/85 (0%)
	Metabolite	10/10 (100%)	0/10 (0%)	8/10 (80%)	0/10 (0%)	8/10 (80%)	0/10 (0%)	8/10 (80%)	0/10 (0%)
Prosocial	Alpha diversity	1/3 (33.33%)	0/3 (0%)	3/3 (100%)	0/3 (0%)	3/3 (100%)	0/3 (0%)	3/3 (100%)	0/3 (0%)
	Relative	49/84 (58.33%)	0/84 (0%)	51/84 (60.71%)	0/84 (0%)	50/84 (59.52%)	0/84 (0%)	49/84 (58.33%)	0/84 (0%)
	Absolute	57/85 (67.06%)	0/85 (0%)	49/85 (57.65%)	0/85 (0%)	48/85 (56.47%)	0/85 (0%)	51/85 (60%)	0/85 (0%)
	Metabolite	6/10 (60%)	0/10 (0%)	5/10 (50%)	0/10 (0%)	5/10 (50%)	0/10 (0%)	5/10 (50%)	0/10 (0%)
**Relative vs Absolute4**									
Internalizing	Child	60/84 (71.43%)	0/84 (0%)	58/84 (69.05%)	0/84 (0%)	56/84 (66.67%)	0/84 (0%)	57/84 (67.86%)	0/84 (0%)
	Mother	59/84 (70.24%)	1/84 (1.19%)	57/84 (67.86%)	2/84 (2.38%)	59/84 (70.24%)	1/84 (1.19%)	58/84 (69.05%)	1/84 (1.19%)
Externalizing	Child	55/84 (65.48%)	0/84 (0%)	61/84 (72.62%)	0/84 (0%)	62/84 (73.81%)	0/84 (0%)	60/84 (71.43%)	0/84 (0%)
	Mother	52/84 (61.9%)	1/84 (1.19%)	47/84 (55.95%)	2/84 (2.38%)	47/84 (55.95%)	1/84 (1.19%)	47/84 (55.95%)	1/84 (1.19%)
Prosocial	Child	59/84 (70.24%)	0/84 (0%)	58/84 (69.05%)	0/84 (0%)	60/84 (71.43%)	0/84 (0%)	63/84 (75%)	0/84 (0%)
	Mother	57/84 (67.86%)	0/84 (0%)	62/84 (73.81%)	0/84 (0%)	64/84 (76.19%)	0/84 (0%)	61/84 (72.62%)	0/84 (0%)

^a^Model 0, *B* ~ *G*; Model 1, *B* ~ *G* + *child sex* + *pubertal status* + *child age* + *food Factor 1* + *food Factor 2*; Model 2, *B* ~ *G* + *child sex* + *pubertal status* + *child age* + *food Factor 1* + *food Factor 2*+ *zBMI*; Model 3, *B* ~ *G* + *child sex* + p*ubertal status* + *child age* + *food Factor 1* + *food Factor 2*+ *zBMI* + *antibiotics* + *diarrhea* + *constipation*; *B* indicating a behavioral measure and *G* indicating a microbial predictor.

^b^Numbers of significant microbiota–behavior relations after FDR correction for multiple tests. *N* = 3 alpha diversity indices, *N* = 84 taxa (for both relative and absolute abundances) prevalent in more than 10% of *N* = 137 participants, *N* = 1 total absolute abundances, *N* = 10 fecal microbiota-derived metabolites.

^c^Similarity of microbiota–behavior relations between using child and maternal reports. *Same direction* indicates that estimates are in the same direction for child and maternal reports. *Same direction and sig* refers to significant estimates after FDR correction that are in the same direction for child and maternal reports.

^d^Similarity of microbiota–behavior relations between using relative and absolute abundances. *Same direction* indicates that estimates are in the same direction between using relative and absolute abundances. *Same direction and sig* refers to significant estimates after FDR correction that are in the same direction for relative and absolute abundances. *N* = 1 total absolute abundance variable was not applicable in this case.

**Figure 2.**
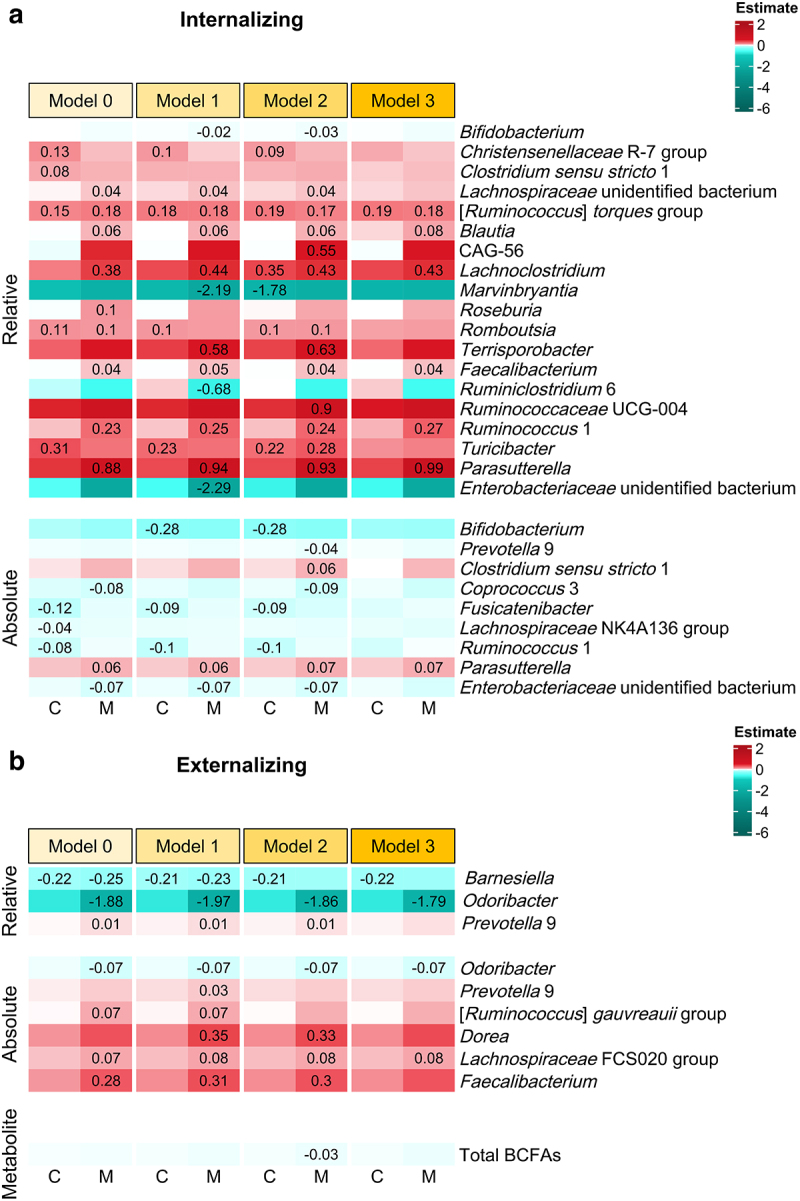
Relations between microbial predictors and behavioral measures, including (a) internalizing and (b) externalizing behavior. The relations that were significant after correction for multiple tests for at least one behavioral measure are displayed. Microbial relations to prosocial behavior are not displayed, due to lack of significant results. Model 0, *B* ~ *G*; Model 1, *B* ~ *G* + *child sex* + *pubertal status* + *child age* + *food Factor 1* + *food Factor 2*; Model 2, *B* ~ *G* + *child sex* + *pubertal status* + *child age* + *food Factor 1* + *food Factor 2*+ *zBMI*; Model 3, *B* ~ *G* + *child sex* + *pubertal status* + *child age* + *food Factor 1* + *food Factor 2*+ *zBMI* + *antibiotics* + *diarrhea* + *constipation*; *B* indicating a behavioral measure and *G* indicating a microbial predictor (i.e., alpha diversity, relative and absolute abundances of genus-level taxa prevalent in more than 10% of *N* = 137 samples, total absolute abundances, and fecal microbiota-derived metabolites). C, child reports; M, maternal reports. The color degree shows the strength of estimates between microbial predictors and behavioral measures. Exact estimate values are shown for significant relations only.

Relative abundances of microbial taxa, including [*Ruminococcus*] *torques* group, *Blautia*, *Lachnoclostridium*, *Faecalibacterium*, *Ruminococcus* 1, and *Parasutterella*, showed positive relations to child internalizing difficulties (in particular maternal reports) across four tested models (Figure 2a). Notably, a higher level of *Parasutterella* absolute abundances was related to more internalizing problems, conforming to results using relative abundances. As for externalizing problems, *Odoribacter* exhibited negative links to maternal reports when using both abundance measures (Figure 2b). Additionally, *Barnesiella* relative abundances were negatively associated with child-reported externalizing difficulties, while absolute abundances of FCS020 group within the *Lachnospiraceae* family were positively related to externalizing problems. Furthermore, we found relative abundances of *Prevotella* 9 positively associated with mother-reported externalizing behavior in all tested conditions except for Model 3 (sensitivity analyses). No significant relations were observed between alpha diversity, total absolute abundances, and behavioral measures. We also did not find significant relations for fecal microbiota-derived metabolites, except for a negative link between total BCFAs and externalizing behavior in Model 2.

#### Child sex-related differences

To test whether child sex-related differences exist in behavioral relations to the microbial predictors, we performed similar GLMs with an extra interaction term consisting of sex (dummy-scored as girl = 0 and boy = 1) and the predictor on the same community samples (complete results in Table S6). As multicollinearity occurred between microbial predictors, child sex, and their interactions in some tested cases (Table S7), these cases were excluded in this study.

For all tested GLMs for which no multicollinearity was observed, up to 18.42% of sex-related differences in microbiota–behavior relations were significant (Table 3). These significant differences were mostly observed between taxon abundances (both relative and absolute) and mother-reported externalizing behavior. Furthermore, more than 40% of sex-related differences were in the same direction for child and maternal reports, but none of them remained significant after correcting for multiple tests. Additionally, only a few sex-dependent differences were found in the same direction and significant for both relative and absolute abundances.

**Table 3.** Summary of child sex-related differences in microbiota–behavior relations.

		Model 1^1^		Model 2		Model 3	
Significant relations^2^		Child	Mother	Child	Mother	Child	Mother
Internalizing	Alpha diversity	NA	NA	NA	NA	NA	NA
	Relative	1/55 (1.82%)	0/46 (0%)	1/55 (1.82%)	0/46 (0%)	2/53 (3.77%)	1/46 (2.17%)
	Absolute	0/35 (0%)	1/28 (3.57%)	0/33 (0%)	0/27 (0%)	0/33 (0%)	2/27 (7.41%)
	Metabolite	0/5 (0%)	0/4 (0%)	0/4 (0%)	0/4 (0%)	0/4 (0%)	0/3 (0%)
Externalizing	Alpha diversity	NA	NA	NA	NA	NA	NA
	Relative	0/48 (0%)	5/42 (11.9%)	0/48 (0%)	5/40 (12.5%)	1/45 (2.22%)	7/38 (18.42%)
	Absolute	0/29 (0%)	2/18 (11.11%)	0/29 (0%)	2/18 (11.11%)	1/29 (3.45%)	3/18 (16.67%)
	Metabolite	0/2 (0%)	0/3 (0%)	0/2 (0%)	0/3 (0%)	0/2 (0%)	0/2 (0%)
Prosocial	Alpha diversity	NA	NA	NA	NA	NA	NA
	Relative	0/47 (0%)	0/48 (0%)	0/47 (0%)	0/48 (0%)	0/47 (0%)	0/48 (0%)
	Absolute	0/28 (0%)	0/30 (0%)	0/28 (0%)	0/30 (0%)	0/28 (0%)	0/29 (0%)
	Metabolite	0/2 (0%)	0/2 (0%)	0/2 (0%)	0/2 (0%)	0/2 (0%)	0/2 (0%)
**Child vs Mother3**		**Same direction**	**Same direction and sig**	**Same direction**	**Same direction and sig**	**Same direction**	**Same direction and sig**
Internalizing	Alpha diversity	NA	NA	NA	NA	NA	NA
	Relative	34/45 (75.56%)	0/45 (0%)	34/45 (75.56%)	0/45 (0%)	33/44 (75%)	0/44 (0%)
	Absolute	21/28 (75%)	0/28 (0%)	22/27 (81.48%)	0/27 (0%)	18/27 (66.67%)	0/27 (0%)
	Metabolite	4/4 (100%)	0/4 (0%)	3/3 (100%)	0/3 (0%)	3/3 (100%)	0/3 (0%)
Externalizing	Alpha diversity	NA	NA	NA	NA	NA	NA
	Relative	32/41 (78.05%)	0/41 (0%)	32/40 (80%)	0/40 (0%)	33/38 (86.84%)	0/38 (0%)
	Absolute	15/18 (83.33%)	0/18 (0%)	15/18 (83.33%)	0/18 (0%)	16/18 (88.89%)	0/18 (0%)
	Metabolite	0/2 (0%)	0/2 (0%)	0/2 (0%)	0/2 (0%)	0/2 (0%)	0/2 (0%)
Prosocial	Alpha diversity	NA	NA	NA	NA	NA	NA
	Relative	22/47 (46.81%)	0/47 (0%)	21/47 (44.68%)	0/47 (0%)	19/47 (40.43%)	0/47 (0%)
	Absolute	17/28 (60.71%)	0/28 (0%)	17/28 (60.71%)	0/28 (0%)	16/28 (57.14%)	0/28 (0%)
	Metabolite	2/2 (100%)	0/2 (0%)	2/2 (100%)	0/2 (0%)	2/2 (100%)	0/2 (0%)
**Relative vs Absolute4**							
Internalizing	Child	27/31 (87.1%)	0/31 (0%)	25/30 (83.33%)	0/30 (0%)	27/30 (90%)	0/30 (0%)
	Mother	18/24 (75%)	0/24 (0%)	17/23 (73.91%)	0/23 (0%)	18/23 (78.26%)	1/23 (4.35%)
Externalizing	Child	20/23 (86.96%)	0/23 (0%)	20/23 (86.96%)	0/23 (0%)	20/22 (90.91%)	0/22 (0%)
	Mother	15/18 (83.33%)	0/18 (0%)	14/17 (82.35%)	0/17 (0%)	16/17 (94.12%)	1/17 (5.88%)
Prosocial	Child	22/24 (91.67%)	0/24 (0%)	22/24 (91.67%)	0/24 (0%)	22/24 (91.67%)	0/24 (0%)
	Mother	19/25 (76%)	0/25 (0%)	18/25 (72%)	0/25 (0%)	17/24 (70.83%)	0/24 (0%)

^a^Model 1, *B* ~ *G* + *child sex* + *pubertal status* + *child age* + *food Factor 1* + *food Factor 2* + *G:child sex*; Model 2, *B* ~ *G* + *child sex* + *pubertal status* + *child age* + *food Factor 1* + *food Factor 2*+ *zBMI + G:child sex*; Model 3, *B* ~ *G* + *child sex* + p*ubertal status* + *child age* + *food Factor 1* + *food Factor 2*+ *zBMI* + *antibiotics* + *diarrhea* + *constipation + G:child sex*; *B* indicating a behavioral measure and *G* indicating a microbial predictor.

^b^Numbers of significant child sex-related differences in microbiota–behavior relations after FDR correction for multiple tests. The numbers after slashes indicate tested conditions without multicollinearity issues. All alpha diversity-included models exhibited multicollinearity problems, and therefore calculating significant relations is not applicable (NA) to them.

^c^Similarity of child sex-related differences in microbiota–behavior relations between using child and maternal reports. *Same direction* indicates that estimates of sex-related differences are in the same direction for child and maternal reports. *Same direction and sig* refers to significant estimates after FDR correction that are in the same direction for child and maternal reports. The numbers after slashes indicate common microbial predictors between non-multicollinearity models using child and maternal reports. These comparisons are not applicable (NA) to alpha diversity due to multicollinearity problems.

^d^Similarity of child sex-related differences in microbiota–behavior relations between using relative and absolute abundances. *Same direction* indicates that estimates of sex-related differences are in the same direction between using relative and absolute abundances. *Same direction and sig* refers to significant estimates after FDR correction that are in the same direction for relative and absolute abundances. The numbers after slashes indicate common microbial predictors between non-multicollinearity models using relative and absolute abundances.

**Figure 3.**
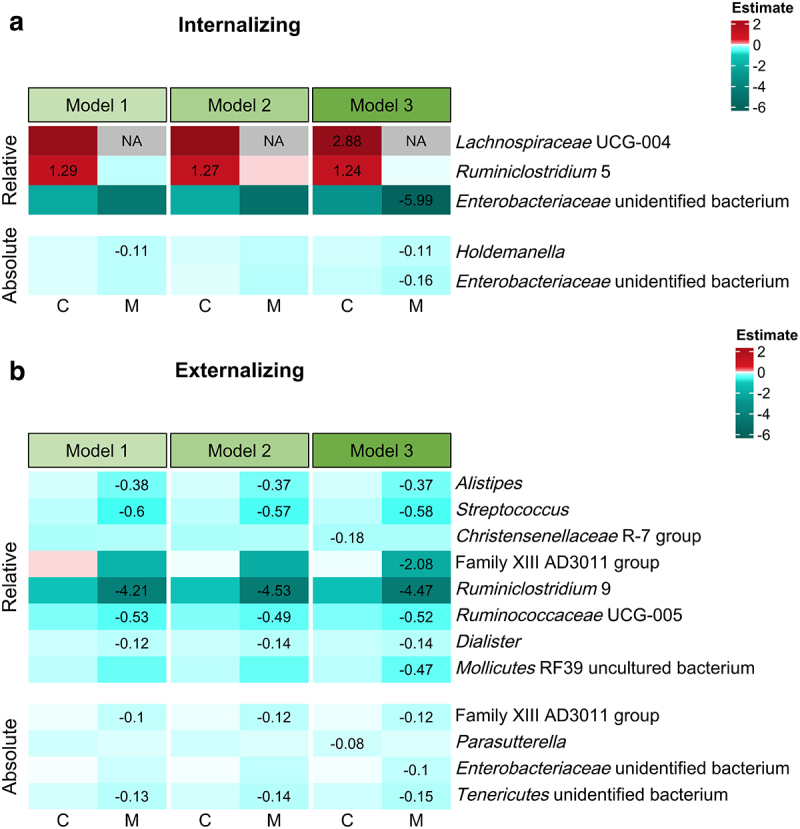
Child sex-related differences in relations between microbial predictors and behavioral measures, including (a) internalizing and (b) externalizing behavior. The differences that were significant after correction for multiple tests for at least one behavioral measure are displayed. Differences related to prosocial behavior are not displayed, due to lack of significant results. Model 1, *B* ~ *G* + *child sex* + *pubertal status* + *child age* + *food Factor 1* + *food Factor 2* + *G:child sex*; Model 2, *B* ~ *G* + *child sex* + *pubertal status* + *child age* + *food Factor 1* + *food Factor 2*+ *zBMI* + *G:child sex*; Model 3, *B* ~ *G* + *child sex* + *pubertal status* + *child age* + *food Factor 1* + *food Factor 2*+ *zBMI* + *antibiotics* + *diarrhea* + *constipation* + *G:child sex*; *B* indicating a behavioral measure and *G* indicating a microbial predictor (i.e., alpha diversity, relative and absolute abundances of genus-level taxa prevalent in more than 10% of *N* = 137 samples, total absolute abundances, and fecal microbiota-derived metabolites). C, child reports; M, maternal reports. The color degree shows the estimates of sex-related differences, with a positive value indicating more behavioral problems in boys (dummy-scored as 1) in response to one-unit increase of a microbial predictor, compared to girls (dummy-scored as 0). Exact estimate values are shown for significant sex-related differences only. NA means not applicable due to multicollinearity issues.

Across the three tested models with microbiota–sex interactions, a higher level of *Ruminiclostridium* 5 relative abundances was consistently related to more child-reported internalizing problems, in boys compared to girls (Figure 3a). Such inter-model consistency also was observed between mother-reported externalizing difficulties and seven microbial taxa (Figure 3b), including *Alistipes*, *Streptococcus*, *Ruminiclostridium* 9, *Ruminococcaceae* UCG-5, *Dialister* (relative abundances), and Family XIII AD3011 group and an unidentified bacterium within *Tenericutes* (absolute abundances). All these taxa were associated with less externalizing behavior in boys compared to girls. Microbiota–behavior relations were not compared between sexes for alpha diversity due to multicollinearity issues. No sex-biased relations were found between fecal microbiota-derived metabolites and behavioral measures.

## Discussion

Our study aimed to examine child sex differences in gut microbiota composition in children at an early stage of puberty, and to explore the associations between their behavior and gut microbiota composition and microbiota-derived fecal metabolites, also contemplating potential sex-related differences in these associations. Regarding the first aim, our results did not reflect sex-specific differences in alpha diversity and genus-level microbial taxon abundances but showed modest sex-dependent variations in beta diversity at this age. As for the second aim, multiple relations were observed between child behavior and taxon relative and absolute abundances, while associations were almost absent between behavior and microbial diversity as well as microbiota-derived fecal metabolites.

Biological sex differences have been observed in adult gut microbiota composition in population-level studies,^3,27^ along with changes in abundances of specific microbial taxa, such as *Prevotella*, *Bifidobacterium*, *Akkermansia*, and *Ruminococcus*.^7–9^ This discrepancy is assumed to most probably be attributed to gonadal hormones.^10^ However, relatively little is known on children in puberty, when multiple simultaneous changes are being initiated in physiology and behavior. Here too, differences in gonadal hormones, as represented by pubertal status, were put forward as a hypothesis, leading to divergence in gut microbiota composition between boys and girls in early puberty.^10^ In our study, we found slight sex-specific variance in beta diversity and no differences in alpha diversity and abundances of individual genus-level microbial taxa (both relative and absolute abundances). In line with this, Falony et al. Reported a significant but small amount of compositional variance (beta diversity) explained by biological sex, in two population-based adult cohorts.^4^ Importantly, our findings on alpha diversity align with those of a previous study in children at around age 11.^11^ Regarding relative abundances, Yuan et al. Found 11 differentially abundant taxa (e.g., *Phascolarctobacterium*, *Parabacteroides*, and *Alistipes*, which were enriched in girls) between sexes in a group of five- to 15-year-old Chinese children, by using Wilcoxon rank sum test.^11^ Furthermore, adult studies reflected apparently higher relative abundances of *Prevotellaceae* taxa in males, and higher levels of *Bifidobacterium*, *Akkermansia*, and *Ruminococcaceae* taxa in females.^8,9^ Taken together, these results suggest that puberty might be the start for the gut microbiota to differentiate between sexes. It awaits to be further explored if these slight microbial differences in early puberty become stronger during later puberty and then stabilize when gonadal hormones reach a steady state in adulthood.

Our analyses on microbiota-behavior associations showed no relations between alpha diversity and behavioral measures after correction for multiple testing. In a study in infants, Carlson et al. showed increased alpha diversity at the age of one year to be associated with lower cognitive ability at the age of two years.^13^ Other studies on infants and pre-schoolers found increased alpha diversity in relation to less internalizing behavior.^15,16^ However, this comparison between infants and preschoolers and pubertal children is not without risks, as in puberty both the gut microbiota and the brain are thought to experience a second period of rapid growth and change after the first years of life, potentially influencing some gut–brain interactions.

In correspondence with previous studies, our results also suggest multiple associations between microbial taxa and child behavior. More than half of these relations were observed to have the same direction between child and maternal reports or between relative and absolute abundances, despite only a minority of them being significant relations. Moreover, the different reporters and abundance types produced a various number of significant relations, depending on the behavioral scales. In the following, we discuss *Parasutterella* and *Odoribacter* for which both relative and absolute abundances exhibited significant relations to at least one behavioral measure. Next, we specifically discuss the relations of *Prevotella* 9, as it has been put forward as a noteworthy microbial taxon in our earlier research.^18^

In the present study, we found that relative and absolute abundances of *Parasutterella* were positively related to mother-reported internalizing behavior. Interestingly, overgrowing *Parasutterella* has been observed in patients with major depressive disorder (MDD).^28^ Furthermore, depressive symptoms might be at least partly attributed to higher proinflammatory cytokines stimulated by lipopolysaccharides from this Gram-negative bacterium.^29^ Recently, Yao et al. Found that the interaction between *Parasutterella* abundances and dietary sugar consumption was modestly associated with less anxious symptoms and alleviated anxiety severity in adults.^30^ Additionally, *Parasutterella* could actively engage in the metabolism of bile acids (critical to digestion and absorption of fats) and tryptophan (an essential amino acid prevalent in dairy products and nuts, and a precursor of serotonin), further emphasizing the importance of dietary effects on child behavior.^31^

Additionally, we found relative and absolute abundances of *Odoribacter* in reverse relations to mother-reported externalizing behavior. In accordance with these findings, *Odoribacter* depletion has been observed in children with ASD^32^ and been related to worse performance in elevated plus maze tests performed on mice with early adversity.^33^ Notably, *Odoribacter* has the genetic potential for producing γ-aminobutyric acid (GABA; a primary inhibitory neurotransmitter).^34^ Reduced cerebral GABA concentrations have been found in children with ADHD^35^ and ASD^36^.

Apart from aforementioned genus-level microbial taxa, another noteworthy relation was found between higher relative abundances of *Prevotella* 9 and more mother-reported externalizing difficulties. This complies with our earlier findings in the same cohort that increased *Prevotella* 9 at ages 6 and years was associated with more externalizing problems at age 10.^18^ Furthermore, *Prevotella* 9 relative abundances at the ages of 6, 10, and 12 years were highly correlated with each other (all Spearman correlation coefficients higher than 0.75, with *p* values lower than 1 × 10^–23^; Table S8). This implies that an early intervention targeting *Prevotella* 9 might have enduring effects on its relative abundances at later ages. Even though the underlying mechanism remains unclear yet, an altered gut inflammatory status might be behind associations between *Prevotella* 9 and externalizing behavior.^37^ Recently, Iljazovic et al. Found that one *Prevotella* species exacerbated gut inflammation in mice, characterized by reduced concentrations of SCFAs and raised levels of pro-inflammatory cytokines.^38^ However, it is important to be aware of the divergent roles that *Prevotella* spp. may play in various scenarios. Additionally, *Prevotella* was observed to be highly prevalent in non-westerners consuming plant-rich diets, and therefore supposed to exert beneficial effects on host health.^39^ Regarding child mental health, Loughman et al. Observed a reduction in *Prevotella* in one-year-old infants related to more internalizing problems at age two.^17^ Putting aside the specifics, previous and current results on relations between *Prevotella* and problem behavior point at this microbial taxon as an interesting target for future studies.

Regarding microbiota-derived fecal metabolites, we did not find any consistent associations to the studied behavior among the different models accounting for different variables. Although this may appear striking, it is worth noting that despite fecal SCFAs being widely recognized as beneficial to general health, their roles in mental health have not been fully determined.^21,23^ For instance, increased propionate may partly underlie the pathology of ASD in some children.^23^ In line with this, an *in vivo* study showed ASD-like symptoms in rats after intracerebroventricular injections of supraphysiological propionate.^40^ Additionally, depression in adults was related to reduced levels of *Oscillibacter* species,^41^ of which the main end product is valerate, structurally resembling GABA. However, follow-up assessments did not show decreased fecal valerate in depressed adults but increased levels of its isomer, isovalerate.^25^ Until now, evidence is limited and less uniform in human research. In addition to being used as energy source by colonic epithelial cells, importantly, SCFAs can activate receptors on immune, enteroendocrine, and vagal nerve cells, indirectly impacting brain physiology and functions via a complex signaling network.^21^ Finally, it is relevant to know that the production of some fecal metabolites, such as the SCFAs and BCFAs investigated in the present study, is largely influenced by the amount, category, and even structure of the dietary ingredients.^42^ This suggests that in future studies it will be essential to more comprehensively record metabolite levels and food consumption, such as by means of 24-h recalls.

Regardless of the growing evidence linking the gut microbiota to child behavior, relatively few investigations have been conducted to examine sex-related differences in these relations.^14,43^ In our study, no identical sex-specific differences were found between relative and absolute abundances of individual taxa, but we observed eight microbial taxa (either relative or absolute abundances) holding consistent inter-model outcomes. These sex-dependent relations were between *Ruminiclostridium* 5 (relative abundances) and child-reported internalizing problems, between *Alistipes*, *Streptococcus*, *Ruminiclostridium* 9, *Ruminococcaceae* UCG-5, *Dialister* (all relative abundances) and mother-reported externalizing difficulties, and between Family XIII AD3011 group, an unidentified bacterium within *Tenericutes* (both absolute abundances) and externalizing behavior reported by mothers. According to a study by Christian et al., surgency (an early temperamental trait that is predictive of subsequent externalizing symptoms^44^ was positively related to alpha and beta diversity as well as *Ruminococcaceae* abundances in boys, and fear reactivity (another early temperamental trait negatively correlated with later externalizing problems^44^ was positively associated with *Rikenellaceae* abundances in girls before age three.^43^ Also Aatsinki et al. Observed sex specificities between microbial taxa with varying compositional features in child temperament.^14^ Additionally, higher relative abundances of *Actinobacteria* were observed in female MDD adults, while lower relative abundances of *Bacteroidetes* were found in male MDD adults.^45^ In sum, our findings and those of previous research accentuate the importance of considering child sex from early puberty onwards as an influential factor on associations between microbiota and behavior. The mechanisms underlying such relations need to be explored comprehensively in forthcoming research.

Our study should be considered with some strengths and limitations. The strengths include: (1) the inclusion of both child and maternal reports; (2) the quantification of the concentrations of several microbiota-derived fecal metabolites, especially SCFAs; (3) the utilization of multiple regression models which considered a range of potential covariates and confounders; and (4) the use of both relative and absolute abundances, emphasizing the necessity to take the total microbial load into account. Our limitations include: (1) due to budget restrictions, 16S rRNA gene sequencing was carried out. This technique is restricted by lower taxonomic resolution, compared with whole-genome shotgun metagenomic sequencing (WGS).^46,47^ WGS enables identifying microbial taxa at the species and strain levels, and this may facilitate the detection of microbiota–behavior relations that cannot be spotted by the 16S rRNA gene based analysis as this is generally restricted in resolution to the genus level; (2) due to financial constraints, only a restricted number of microbiota-derived fecal metabolites were analyzed. Future inclusion of other previously reported relevant metabolites, such as serotonin and GABA, or the use of non-targeted metabolomic approaches to profile all fecal metabolites, is recommended. Moreover, compared to feces, peripheral blood samples (unavailable in the current cohort) are thought to reflect biologically meaningful metabolite levels more straightforwardly; (3) primer bias may occur during qPCR^48;^ (4) the food frequency questionnaire provides fewer details of daily food consumption than other instruments; (5) causal relationships cannot be determined in a cross-sectional observational study; and (6) our findings should be replicated in similar studies with careful thought of potential covariates and confounders.

In conclusion, subtle and slight differences were observed in gut microbiota composition between boys and girls at an early stage of puberty. Whether these differences remain stable or become larger in adolescence remains to be determined. Gut microbiota composition was associated with problem and prosocial behavior in this low-risk community cohort, in a sex-specific manner in some cases. Finally, this study validated our earlier findings in middle childhood that *Prevotella* 9 levels were positively associated with externalizing behavior. Considering the scarcity of investigations on the gut microbiota–behavior relations in low-risk pubertal populations, our study adds novel and relevant results that can serve as a basis for future research in this field.

## Materials and methods

### Study subjects

The study included low-risk children (*N* = 137) aged around 12 years (12.7 ± 0.3) from the ongoing longitudinal Dutch study named BIBO (Basale Invloeden op de Baby Ontwikkeling; *N* = 193 originally recruited in pregnancy),^49^ with approval from the ethical committee of the Faculty of Social Sciences of Radboud University (ECG300107, ECG13012012, SW2017–1303–497 and SW2017–1303–498). The original recruitment criteria and procedures are described elsewhere.^49^ Characteristics of the current sample are presented in Table 1. The present study was preregistered on the Open Science Framework via this link https://osf.io/wu2vt.

### Procedures of data collection

Child stool samples were collected in sterilized plastic screw-top tubes by either children or their parents immediately after defecation. Before stool collection, instructions were provided to each family. All families were asked to collect stool samples by using the scoop attached to the tube cap. Three scoops were required to ensure sufficient volume for analyses. The filled tube was then placed in a supplied zip-lock bag and immediately kept in home freezers at −20°C for temporary storage. The fecal samples were delivered to the lab by parents or picked up by researchers at the home. During transportation, the fecal samples were placed in cooler bags with cooling elements to keep frozen. Upon arrival at the lab, the fecal samples were long-term stored at −80°C prior to being processed. Children as well as their mothers were asked to fill in online questionnaires separately by using personal links. In two cases, fathers filled in the questionnaire, as the mothers were unavailable during the data collection period. For an easy interpretation, they were still called maternal reports and included in the study. The questionnaires filled in by both children and mothers pertained behavior. Additionally, children filled in questionnaires about diet and pubertal status, and mother completed questionnaires about child health and demographics.

### Measures

#### Gut microbiota composition

Briefly, DNA was extracted from 0.01 to 0.13 g of fecal samples by using the Maxwell 16 Total RNA system (Promega, Wisconsin, USA) with Stool Transport and Recovery Buffer (STAR; Roche Diagnostics Corporation, Indianapolis, IN), as described previously.^50^ The V4 region of 16S rRNA gene of bacteria and archaea was amplified for each sample in duplicate to reach sufficient concentration for purification. Then, purified amplicons were adjusted to 200 ng each sample before being sequenced, by following steps delineated earlier.^18^ Amplicon sequence variants (ASVs) were identified from 16S rRNA gene sequence data through *NG-Tax* 2.0 (i.e., a semantic framework for high-throughput analysis and classification of marker gene amplicon sequences including the 16S rRNA gene of bacteria and archaea. This framework can be applied to different types of reads (single or merged; paired-end or not). *NG-Tax* 2.0 had a comparable performance with QIIME 2 pipeline plugin DADA2.).^51,52^ ASVs were assigned to taxa referring to SILVA_132_SSU 16S rRNA gene reference database.^53^ Subsequently, we obtained a total of 32,081,185 reads with median reads of 226, 625 per sample.

To obtain copy numbers of the 16S rRNA gene (total bacteria and archaea) within individuals, qPCR reactions were performed in triplicate as follows: (1) 5 μl of SYBR Green Master Mix, 2.6 μl of nuclease-free water, 0.2 μl of 331F universal primer (5’-TCCTACGGGAGGCAGCAGT), 0.2 μl of 797 R universal primer (5’-GGACTACCAGGGTATCTAATCCTGTT),^54^ and 2 μl of 1 ng/μl templates (in standard reactions, full-length 16S rRNA gene amplicons of *Escherichia coli*, diluted to 10^8^ to 10^1^ copy numbers/μl) were used for each reaction; (2) the qPCR program included 10 min of initial denaturation at 95°C and 40 quantification cycles, consisting of denaturation for 15 s at 95°C, annealing for 30 s at 60°C, and elongation for 15 s at 72°C. The raw data were then pre-processed by the CFX Maestro Software.

#### Fecal metabolites

The supernatant of the mixture of 0.2 g fecal sample and 800 µL demineralized water was treated with Carrez reagents to remove protein.^55,56^ Then, the deproteinized supernatant was analyzed by high performance liquid chromatography (HPLC; Shimadzu LC-2030C Plus), equipped with refractive index and UV light (210 nm) detectors. The separation was completed on a Shodex SH1011 column with a flow rate of 1 mL/min at 45°C. The eluent was 0.01 N sulfuric acid. Ten µL of sample supernatant was injected into the column. The concentrations of fecal lactate, acetate, propionate, isobutyrate, butyrate, isovalerate, valerate, were determined according to the area under peaks as depicted and analyzed by Chromeleon^TM^ Chromatography Data System (CDS) Software. The standard series of these metabolites included 0.1, 0.2, 0.3, 1, 2, 3, 10, 20, 30 mmol/L. Additionally, we calculated total SCFAs (including acetate, propionate, and butyrate), total BCFAs (including isobutyrate and isovalerate), and the ratio of total BCFAs to total SCFAs. Among these tested metabolites, SCFAs are mainly produced through the fermentation of dietary fiber, while BCFAs are commonly formed through the fermentation of protein in the distal large intestine.^23,24^ The ratio of total BCFAs to total SCFAs was used as an indicator of relative differences between protein and fiber fermentation. Acetate, propionate, butyrate, valerate, and total SCFAs were positively correlated with each other, and isobutyrate, isovalerate, total BCFAs, and the ratio of total BCFAs to total SCFAs were also positively inter-correlated (Spearman correlations in Figure S1).

#### Behavioral measures

To assess problem behavior (i.e., internalizing and externalizing behavior) and prosocial behavior, children and mothers were asked to fill in the Strengths and Difficulties Questionnaire (SDQ).^57^ Internalizing behavior refers to behavioral problems that influence internal psychological conditions, such as depression, anxiety, somatic states, and social withdrawal, whereas externalizing behavior is manifested as outward behavior, such as aggression, acting out, hyperactivity, hostility, and antisocial behavior. Prosocial behavior is interpreted as an intention to voluntarily help and benefit others. Higher scores on the internalizing and externalizing scales reflect difficulties, while higher scores on the prosocial scale indicate strengths. To obtain a panoramic view of the child’s behavior, both self-report and maternal report data were collected.

Internal consistency of the scales was confirmed by ω_total_ estimates,^58^ computed by the *psych* R package.^59^ Most of the estimates were larger than 0.7 (Table S9), reflecting acceptable internal consistency and indicating that the scales were reliable. Only the child prosocial behavioral scale showed a questionable estimate, but this conformed to previous Dutch research,^60^ and thus was included in the current study. Internalizing, externalizing, and prosocial behavioral scales were positively correlated between child and maternal reports with Spearman correlation coefficients of 0.55, 0.51, and 0.24, respectively (Figure S2).

#### Additional variables

In addition to the child sex (girl or boy),^19,61^ we considered the following variables as potential confounders,^4,62,63^ which may influence both the microbial predictor and the behavioral outcome: (1) Pubertal status was measured by self-reported Tanner stages.^64^ This status was calculated as the average of thelarche (or testicular) development and pubarche, leading to a final score ranging from one (a prepubertal status) to five (a complete sexual maturity). Among 135 children providing puberty information at ages 12 and 14, 97% of them (131/135) reported normal development, and only 3% (4/135) reported reversals (i.e., lower scores at age 14). To reduce bias in reversed conditions, we used average scores at ages 12 and 14 instead of scores at age 12. Note that pubertal status was not described in our preregistration but included here to more precisely delineate sex-related differences in either gut microbiota or its relations to behavior in puberty; (2) child age in years (one missing value was identified among 137 samples, and replaced with the average value of the remaining available data); (3) two food factors (i.e., Factor 1: healthy foods; Factor 2: snacks) based on a 25-item food frequency questionnaire (Table S10), scored on a seven-point scale and collected during the online questionnaire fill-in procedure.

Besides, zBMI was treated as a potential covariate of the gut microbiota,^65^ and a potential intermediate between diet and behavior.^66,67^ It was calculated from child height, weight, child sex, and age according to the WHO Growth Reference via the *zscore* R package (missing values were processed as described in Supplementary materials).^68^

Additionally, three potential covariates of the gut microbiota (i.e., variables considered to only impact the microbial predictor),^12,69^ were collected by a child health questionnaire,^70^ during the online questionnaire fill-in procedure: Whether a child (1) took antibiotics, (2) had diarrhea, and (3) had constipation in the past one year. These variables were dummy-scored as no = 0 and yes = 1.

### Statistical analyses

All analyses were performed in R studio (version 4.1.1; this version is an update of the version 3.6.1 that was described in the preregistration).

#### Demographics and descriptives

We compared variables (including pubertal status, age in years, food Factor 1, food Factor 2, zBMI, antibiotics, diarrhea, constipation, and behavioral measures) between boys and girls by using Wilcoxon tests (for numeric variables) and Chi-square tests (for categorical variables). Behavioral measures were compared between reporters by Wilcoxon tests.

#### Gut microbiota data transformation

Although relative abundance data are frequently and widely used in human studies to describe gut microbiota composition, it has been pointed out that such data come with inherent limitations, including high false discovery rates and more correlational biases.^48,71^ Therefore, we included both relative (0–100%) and absolute abundances (counts per gram of wet feces; calculation procedures are described in Supplementary materials) at the genus level for each sample in this study. Total absolute abundance (total counts per gram of wet feces) in each sample was calculated by summing taxon absolute abundances in each sample.

#### First aim: child sex-related differences in gut microbiota composition

We explored if sex-related differences could exist in three characteristics of gut microbiota composition: (1) alpha diversity, including Chao1, Shannon, and phylogenetic diversity, calculated based on ASV count data by using the *ape* and *picante* R packages;^72,73^(2) beta diversity, including relative abundance-based Bray-Curtis dissimilarity matrix and absolute abundance-based Aitchison distance matrix, computed by the *vegan* and *phyloseq* R packages;^74,75^ (3) individual genus-level microbial taxa prevalent in more than 10% of all subjects, for both relative abundances and log-transformed absolute abundances (log-transformed total absolute abundances were also compared between sexes as an extension of individual taxa).

Specifically, we carried out generalized linear models (GLMs; by *MASS* R package^76^ to explore sex-related differences in alpha diversity, and relative and absolute abundances of individual taxa (Wilcoxon tests as described in the preregistration were not used mainly due to limitations in accounting for potential covariates of the gut microbiota). The distribution normality of these microbial variables was determined based on Shapiro – Wilk tests, with *p* more than 0.05 indicating normal distribution. Gaussian distribution was used for normally-distributed variables, while negative binomial distribution was used for non-normally distributed continuous variables (Table S11).^77^ For beta diversity, we conducted redundancy analysis (RDA) and assessed the variance (R^2^%) explained by child sex after the variance explained by gut microbiota covariates was taken out.

A formula considering covariates of the gut microbiota was used in both GLMs and RDA: *G* ~ *child sex* + *pubertal status* + *child age* + *food Factor 1* + *food Factor 2* + *zBMI* + *antibiotics* + *diarrhea* + *constipation* (*G* indicates alpha diversity, beta diversity, or abundances of individual taxa).

#### Second aim: associations between the gut microbiota and internalizing, externalizing, and prosocial behavioral measures

Through RDA, we detected to what magnitude the variance in gut microbiota beta diversity (either relative abundance-based Bray-Curtis dissimilarity matrix or absolute abundance-based Aitchison distance matrix) was explained by behavioral measures. Two types of variances were included: (1) variance explained by solely one behavioral measure without considering any other variables (formula *G* ~ *B*; *G* indicates a beta diversity matrix and *B* refers to a behavioral measure); (2) when the simple variance explained by a behavioral measure was observed with *p* < .05, we measured its variance after taking out variance explained by potential covariates (formula *G* ~ *B + child sex* + *pubertal status* + *child age* + *food Factor 1* + *food Factor 2* + *zBMI* + *antibiotics* + *diarrhea* + *constipation*).

Then, we performed GLMs, which were also included in another study focused on the same child cohort,^78^ to analyze if each behavioral measure could be predicted by gut microbiota composition (i.e., alpha diversity, and relative abundances and log-transformed absolute abundances both at the genus level) or microbiota-derived fecal metabolites (i.e., SCFAs, BCFAs, lactate, total SCFAs, total BCFAs, and the ratio of total BCFAs to total SCFAs). As none of the behavioral measures were normally distributed (Table S11), we used Poisson distribution for these discrete variables.^77^ To avoid over-sparsity and retain more microbial taxa, we performed the models on taxa prevalent in more than 10% of all subjects. Several different models were conducted as follows:

1. **Model 0** *B*_*i*_ *~ G*_*j*_ was used to measure the independent relation between the outcome variable (*B*_*i*_ is the matrix of behavioral measures, with “*i*” indicating one measure assessed either by child or maternal reports) and the predictor (*G*_*j*_ is the matrix of alpha diversity, microbial abundances, and fecal metabolites, with “*j*” being a diversity parameter, a taxon, or a metabolite);
2. According to the principals based on a causal diagram, namely the directed acyclic graph,^79^ we accounted for potential confounders in **Model 1** *B*_*i*_ *~ G*_*j*_ *+ child sex + pubertal status + child age + food Factor 1 + food Factor 2*;
3. To remove the potential intermediate effect of zBMI between diet and behavior, we additionally included it in **Model 2** *B*_*i*_ *~ G*_*j*_ *+ child sex + pubertal status + child age + food Factor 1 + food Factor 2 + zBMI*;
4. Antibiotics, diarrhea, and constipation were regarded as covariates of microbial predictors only as described above, and thus were treated as neutral variables unnecessary to account for.^79^ However, since these variables have been included in other mental health-related studies, we performed sensitivity analyses to test the consistency between our models by using **Model 3** *B*_*i*_ *~ G*_*j*_ *+ child sex + pubertal status + child age + food Factor 1 + food Factor 2 + zBMI + antibiotics + diarrhea + constipation*.

To explore child sex-related differences in the associations between the gut microbiota and behavioral measures, we added an additional interaction term of child sex and the predictor to the models displayed earlier:

1. **Model 1 with the interaction** *B*_*i*_ *~ G*_*j*_ *+ child sex + pubertal status + child age + food Factor 1 + food Factor 2 + G*_*j*_*: child sex*;
2. **Model 2 with the interaction** *B*_*i*_ *~ G*_*j*_ *+ child sex + pubertal status + child age + food Factor 1 + food Factor 2 + zBMI + G*_*j*_*: child sex*;
3. **Model 3 with the interaction** *B*_*i*_ *~ G*_*j*_ *+ child sex + pubertal status + child age + food Factor 1 + food Factor 2 + zBMI + antibiotics + diarrhea + constipation + G*_*j*_*: child sex*.

#### Multicollinearity

Multicollinearity strengths between variables (on the right side of GLM formulas) were assessed by the variance inflation factor (VIF; by *car* R package^80^. VIF values larger than three indicate multicollinearity issues,^81^ and their corresponding cases were excluded from this study.

#### Significance

For multiple Wilcoxon and Chi-square tests as well as GLMs, *p* values were corrected by the FDR method, with adjusted *p* lower than 0.1 accepted as significant. For RDA, the significance was determined as *p* lower than 0.05 in permutation tests (*N* = 1000).

## Supplementary Material

Supplemental Material

## Data Availability

As the findings in this study are supported by datasets from an ongoing longitudinal cohort, these datasets currently cannot be made publicly available but are available upon request from C.de.W. (Carolina.deWeerth@radboudumc.nl) R codes and necessary result data used for making the heatmap figures are available online https://doi.org/10.5281/zenodo.7881878.
