# Supplementary material for: Gut microbiota and child behavior in early puberty: does child sex play a role?
Source: Gut Microbes. 2023 Nov 9;15(2):2278222. doi: 10.1080/19490976.2023.2278222 (PMC10731618; doi:10.1080/19490976.2023.2278222)
Supplement: Supplemental Material [file KGMI_A_2278222_SM3959.zip › BIBO_12y_SuppFigures_clean.docx]

# Supplementary figures

**Figure S1.** Spearman correlations between concentrations of microbiota-derived fecal metabolites across all subjects. Ratio indicates the ratio of total BCFAs (including isobutyrate and isovalerate) to total SCFAs (including acetate, propionate, and butyrate). The distribution of each metabolite is displayed on the diagonal. Bivariate scatter plots of every two metabolites are shown on the bottom of the diagonal, with a fitted regression line in green. Correlation coefficients are shown on the top of the diagonal, plus their significances represented with asterisks (*, *p* < 0.05; **, *p* < 0.01; ***, *p* < 0.001). Scales along x and y axes indicate metabolite concentrations (or the ratio of total BCFAs to total SCFAs).

**Figure S2.** Spearman correlations between the SDQ scales. The distribution of each behavioral measure is displayed on the diagonal. Bivariate scatter plots of every two behavioral measures were shown on the bottom of the diagonal, with a fitted regression line in green. Correlation coefficients are shown on the top of the diagonal, plus their significances represented with asterisks (*, *p* < 0.05; **, *p* < 0.01; ***, *p* < 0.001). Scales along x and y axes indicate behavioral scores. SDQ: the Strengths and Difficulties Questionnaire; C: child; M: maternal.
