# Supplementary material for: Gut microbiota and child behavior in early puberty: does child sex play a role?
Source: Gut Microbes. 2023 Nov 9;15(2):2278222. doi: 10.1080/19490976.2023.2278222 (PMC10731618; doi:10.1080/19490976.2023.2278222)
Supplement: Supplemental Material [file KGMI_A_2278222_SM3959.zip › BIBO_12y_SuppMaterials_clean.docx]

# Supplementary materials

## Imputation of missing values in zBMI

For a total of 43 participants who did not provide height and weight information at the studied age, we performed imputations following these equations: (1) when height and weight information was provided at both earlier and later ages (i.e., ten and 13 years, N=35), height and weight values for the current study were calculated based on y_height_ = a * x_Age_ + b and y_weight_ = a * x_Age_ + b, where a is the slope and b is the intercept obtained from the linear equation of early and later ages, while x_Age_ indicates the age in days for the present study; (2) when height and weight values were recorded at either age ten years or age 13 years (N=8), the available data are directly used to calculate zBMI for the present study without imputation.

## Calculation procedures of genus-level taxon absolute abundances

Absolute abundance (counts/g wet feces) = $\frac{aVcx}{10bd}$

1. Original fecal DNA concentration: $a$ ng/μl
2. Total volume of original fecal DNA solution: $V$ μl
3. Weight of feces used in fecal DNA isolation: $b$ g wet feces
4. Taxon relative abundance: $c$
5. 16S rRNA gene copy-number variation (referring to the rrnDB database): $d$
6. Copy numbers of qPCR-measured 16S rRNA gene (total bacteria and archaea): $x$ copy numbers/μl (equivalent to $x$ copy numbers/ng DNA, as 1 ng/μl DNA was used in qPCR)
7. The constant term $\frac{1}{10}$ is the dilution factor in fecal DNA isolation through Maxwell
